# Accuracy of keyless vs drill-key implant systems for static computer-assisted implant surgery using two guide-hole designs compared to freehand implant placement: an in vitro study

**DOI:** 10.1186/s40729-023-00470-6

**Published:** 2023-02-07

**Authors:** Clemens Raabe, Tabea S. Schuetz, Vivianne Chappuis, Burak Yilmaz, Samir Abou-Ayash, Emilio Couso-Queiruga

**Affiliations:** 1 Department of Oral Surgery and Stomatology, School of Dental Medicine, University of Bern, Bern, Switzerland; 2 Department of Reconstructive Dentistry and Gerodontology, School of Dental Medicine, University of Bern, Bern, Switzerland; 3 Department of Restorative, Preventive and Pediatric Dentistry, School of Dental Medicine, University of Bern, Bern, Switzerland; 4 Division of Restorative and Prosthetic Dentistry, The Ohio State University College of Dentistry, Columbus, OH USA

**Keywords:** Dental implants, Single tooth, Clinical decision-making, Image-guided surgery, Tooth extraction, Alveolar ridge

## Abstract

**Purpose:**

This in vitro study aimed at comparing the accuracy of freehand implant placement with static computer-assisted implant surgery (sCAIS), utilizing a keyless and a drill-key implant system and two guide-hole designs.

**Methods:**

A total of 108 implants were placed in 18 partially edentulous maxillary models simulating two different alveolar ridge morphologies. 3D digital deviations between pre-planned and post-operative implant positions were obtained. Guide material reduction was assessed in the keyless implant system for the manufacturer’s sleeve and sleeveless guide-hole designs.

**Results:**

sCAIS using a sleeveless guide-hole design demonstrated smaller mean angular, crestal and apical deviations compared to sCAIS utilizing a manufacturer’s sleeve and the freehand group (2.6 ± 1.6°, vs 3.3 ± 1.9°, vs 4.0 ± 1.9°; 0.5 ± 0.3 mm, vs 0.6 ± 0.3 mm, vs 0.8 ± 0.3 mm; and 1.0 ± 0.5 mm, vs 1.2 ± 0.7 mm, vs 1.5 ± 0.6 mm). Smaller angular and apical mean deviations were observed in the keyless implant system as compared with the drill-key implant system (3.1 ± 1.7°, vs 3.5 ± 1.9°, *p* = 0.03; and 1.2 ± 0.6 mm, vs 1.4 ± 0.7 mm, *p* = 0.045). Overall, smaller angular, crestal, and apical deviations (*p* < 0.0001) were observed in healed alveolar ridges (2.4 ± 1.7°, 0.5 ± 0.3 mm, and 0.9 ± 0.5 mm) than in extraction sockets (4.2 ± 1.6°, 0.8 ± 0.3 mm, and 1.6 ± 0.5 mm). Higher mean volumetric material reduction was observed in sleeveless than in manufacturer’s sleeve guide-holes (− 0.10 ± 0.15 mm^3^, vs − 0.03 ± 0.03 mm^3^, *p* = 0.006).

**Conclusions:**

Higher final implant positional accuracy was observed in sCAIS for the keyless implant system, with a sleeveless guide-hole design, and in healed ridges. Sleeveless guide holes resulted in higher volumetric material reduction compared with the manufacturer’s sleeve.

## Background

Dental implant therapy is a favorable treatment option to replace missing teeth using fixed and removable prosthetic restorations [1]. To ensure an esthetic, functional, cleansable, and screw-retained restoration, a “prosthetically driven” implant position respecting the local anatomy is crucial [2]. To achieve the correct 3D implant position, free-handed implant placement orienting on well-established landmarks or using conventional templates such as vacuum-formed stents had been considered the gold standard for decades. However, implant placement using free-handed protocols may result in pronounced deviations between planned and achieved implant positions [3].

To further improve the accuracy of dental implant positioning, digital treatment planning and static computer-assisted implant surgery (sCAIS) were introduced over the years [4–12]. Recent literature reflects the growing body of evidence for improving implant placement accuracy when using sCAIS compared to free-handed protocols in the replication of pre-planned implant position [4–8]. Additionally, digital workflows may enhance clinical protocols by prefabrication of restorations, reduction of overall chair time, and higher patient preference compared to conventional workflows [13, 14]. Therefore, sCAIS has become a popular modality of treatment among clinicians, gaining a critical role in challenging clinical scenarios.

However, although these developments are promising, digital workflow is technique-sensitive and various elements influence the reliability and accuracy of sCAIS [15]. Recent in vitro studies demonstrated differences in the accuracy of sCAIS within and between implant systems [16–18]. These differences may be related to the macro-design of the implants, and the variations in design or tolerances of components, such as surgical drills, drill keys, and guide-hole sleeves used for guided implant site osteotomy and placement. To enhance the accuracy of sCAIS, alternative implant systems or surgical guide design options focusing on the reduction of components, such as keyless compared to drill-key systems or sleeveless (SL) versus manufacturer’s sleeve [19] guide-hole designs have been introduced to the market [18]. Interestingly, recent studies have reported higher accuracy of final 3D implant position when SL guide-hole designs were used [19, 20]. However, the evidence regarding the accuracy of keyless compared to drill-key implant systems is scarce.

As demonstrated in different in vitro and clinical studies, progressive atrophy of the alveolar ridge takes place after the tooth has been extracted from its alveolus, modeling the alveolar ridge over time [21–25]. Therefore, the alveolar ridge morphology, management of the extraction site, and timing of implant placement may also be contributing factors influencing the accuracy and feasibility of implant placement [26–29]. Most recently, in vitro studies demonstrated higher accuracy in the final implant position in fully healed alveolar ridge sites as compared to fresh extraction sockets [30, 31].

There is a lack of evidence regarding the accuracy of free-handed implant placement compared to the use of sCAIS-systems (i.e., drill-key, and keyless designs) including different guide-hole designs (i.e., SL, and MS). Hence, this in vitro study primarily aimed at comparing the accuracy of free-handed and static computer-assisted implant placement in clinical scenarios simulating immediate and delayed single implant placement utilizing two implant systems and two guide-hole designs. The secondary aim of this study was to evaluate guide material reduction in the keyless implant system. Finally, the null hypothesis was that the implant system (H0_1_), the guide hole design (H0_2_), and the alveolar ridge morphology (H0_3_) would not affect the accuracy of implant placement.

## Methods

This in vitro study was conducted in the Department of Oral Surgery and Stomatology at the University of Bern, Switzerland, between November 2021 to February 2022.

### Model selection and preparation

**Fig. 1:**
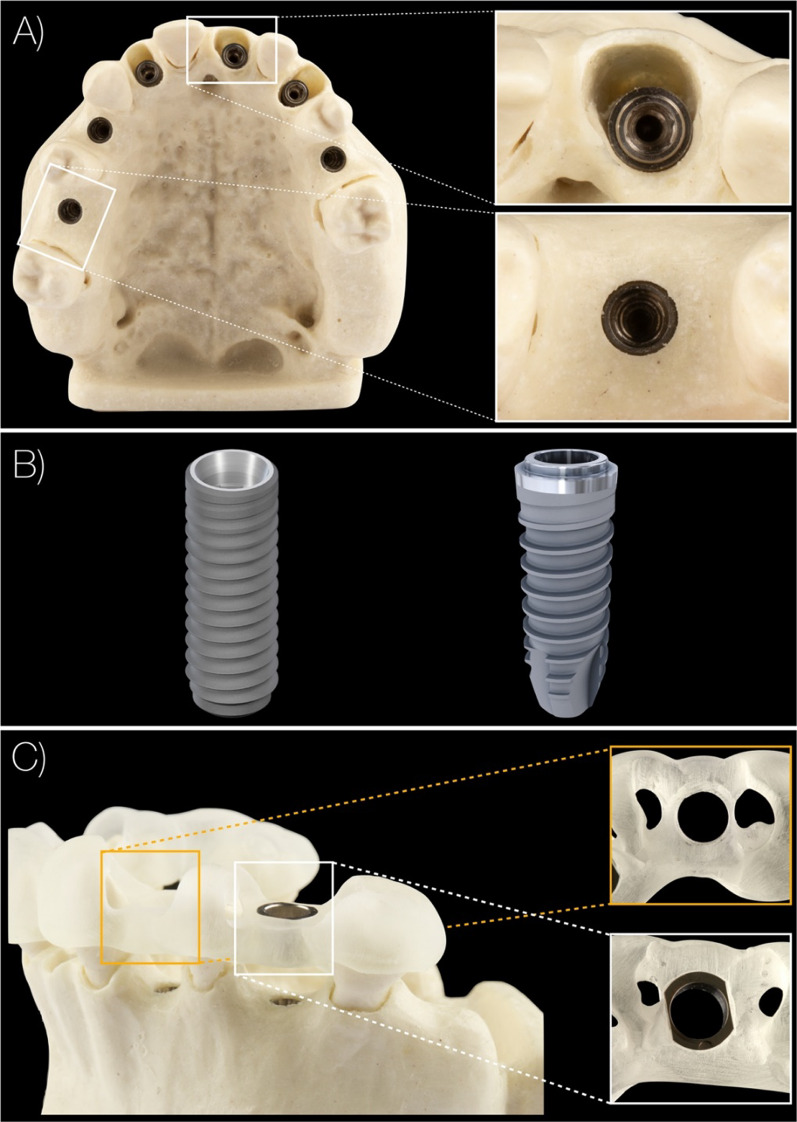
Representative case of a model with implants placed in fresh extraction sockets and healed ridges. Different alveolar ridge morphologies (**A**); implants of the drill-key (left) and keyless (right) systems (**B**). Sleeveless sites (orange); standard manufacturer’s sleeve (white) (**C**)

Partially edentulous maxillary models, simulating natural bone density D2 with a cortico-spongious architecture (BoneModels, Castellón de la Plana, Spain), were utilized in this study. For models simulating clinical scenarios with fresh extraction sockets, single tooth gaps were located at FDI 12, 21, and 23 positions; whereas, for clinical scenarios simulating a fully healed ridge, single tooth gaps were at tooth locations 16, 14, and 25 positions (Fig. 1). Prior to implant placement, a cone beam computed tomography (CBCT) scan (8 × 5 cm, 80 μm voxel size, 90 kVp, 1 mAs; 3D Accuitomo 170, J. Morita Corp, Osaka, Japan) and a surface scan using a laboratory scanner (3Shape 4, 3Shape Inc, Copenhagen, Denmark) was taken. The resulting DICOM and STL files, including a pre-designed digital wax-up made by an experienced dental technician using computer-aided design (CAD) software (Zirkonzahn.Modellier, Zirkonzahn GmbH, Gais, Italy), were imported to an implant planning software (coDiagnostiX, version 10.5, Dental Wings Inc, Montreal, Canada). One team member (C.R) planned 3D implant positions in each designated implant site to support screw-retained single implant crowns.

### Implant systems

Two bone-level type dental implants with similar implant macro-design were used in this investigation: parallel-walled and self-tapping implants with a shallow thread depth and a thread pitch of 0.8 mm (ST: Bone Level 4.1 × 12 mm RC, Straumann AG. Basel, Switzerland) and 1 mm (TH: Element MC 4 × 12.5 mm Thommen SPI, Grenchen, Switzerland) as shown in Fig. 1.

**Fig. 2:**
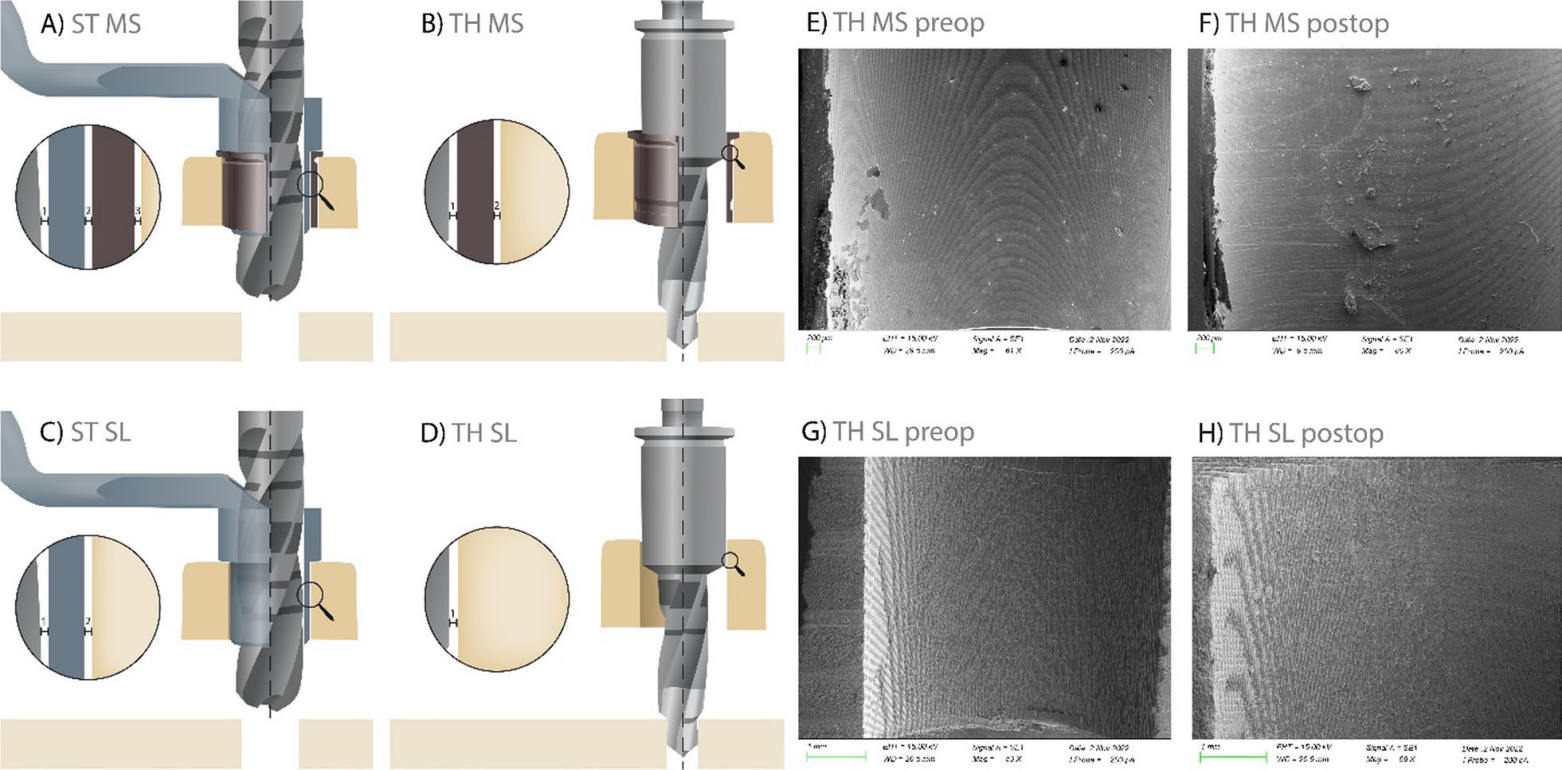
Guide-hole designs: manufacturer sleeve sites for the drill-key system (**A**) including three gaps between drill, key, sleeve, and guide material; and the keyless system (**B**) including two gaps between drill, sleeve, and guide material. Sleeveless sites for the drill-key system (**C**) including two gaps between drill, key, and guide material; and the keyless system (**D**) including one gap between drill and guide material. Visualization of the keyless TH guide-hole deterioration via scanning electron microscope images representing pre- and post-operative sites for MS (**E**, **F**) and SL (**G**, **H**). *ST* drill-key Straumann, *TH* keyless Thommen, *MS* manufacturer’s sleeve, *SL* sleeveless sites

The guide-related surgical components recommended by the manufacturers include the following: surgical drills, guided by a drill key inserted into the MS (three components in total, ST), or surgical drills with drill-integrated sleeve guidance inserted into the MS (two components in total, TH) (Figs. 1, 2). The MS had an inner diameter of 4.8 mm (TH) and 5.0 mm (ST), while the height of the sleeve (5 mm) and material (stainless steel) were equal for both manufacturers.

### Implant placement procedures and surgical guide design

Implant placement procedures included (1) free-handed protocols (FH) with the use of a surgical caliper and implant planning software for intra-operative orientation; (2) sCAIS with surgical guides using MS (stainless steel; Fig. 2a, b); and (3) sCAIS with surgical guides incorporating MS-dimensions to the surgical guide design, resulting in a sleeveless surgical guide (SL; Fig. 2c, d). For both the MS and the SL group, a guide-hole calibration matrix with various offsets was tested independently by three experienced investigators with experience in sCAIS to define the offset for an adequate press fit of the sleeve [19] or surgical instruments (SL) (ST: ± 0.02 mm, TH: ± 0.04 mm). The sleeves [19] were not glued into the surgical guide. To reduce confounding factors by free drilling distance (FDD = implant length + sleeve-to-bone distance) or height of instrument guidance (HIG) as given by the sleeve (TH) or the drill-key within the sleeve (ST) [32], these values were equalized to the greatest extent [Straumann: FDD 18 mm (12 + 6 mm), HIG 6 mm; Thommen: FDD 17.5 mm (12.5 + 5 mm), HIG 5 mm]. The guide material thickness was set to 3.5 mm and the guide-to-teeth offset to 0.15 mm. Multiple fenestrations were created to allow visualization of the guide’s intra-operative fit on the model. For each model, an individual surgical guide was manufactured using a transparent, light-cured resin for stereolithography (ProArt Print Splint, Ivoclar Vivadent AG, Schaan, Lichtenstein) in a 3D printer (PrograPrint PR5, Ivoclar Vivadent AG, Schaan, Lichtenstein) by the same dental technician. For implant placement, the models were mounted in a phantom head. One experienced and board-certified oral surgeon (C.R) performed all implant placement procedures following the manufacturer’s recommendations using a surgical motor (iChiropro, Bien Air, Bienne, Switzerland). Each of the guide-holes in the surgical guides was used for a single implant placement procedure.

### Deterioration assessment of the surgical guides

Potential deterioration of each of the MS and SL sites caused by the drill-integrated sleeve guidance for the keyless implant system was assessed by measuring the mass of the surgical guide before and after implant placement. Therefore, a high accuracy and precision laboratory balance with a readability capacity of 0.0001 g was used (Sartorius Research R180D. Göttingen, Germany). The change in mass of the guide was used to calculate the volume loss of each MS or SL site utilizing the following formula: *V* = *m*/*p*; (*V* = volume, *m* = mass, *p* = density). The density value for TH steel is 8.00 g/cm^3^, whereas the density value of polymerized ProArt Print Splint is 1.14 g/cm^3^. The density values were obtained from the manufacturers.

### Study groups

Three different implant placement protocols (FH, MS, SL) were evaluated for both implant systems (ST, TH) in sites with fresh extraction sockets or healed site morphology. In each model, the groups were randomly assigned in a rotation order to obtain an equally distributed sample size.

### Digital measurements

**Fig. 3:**
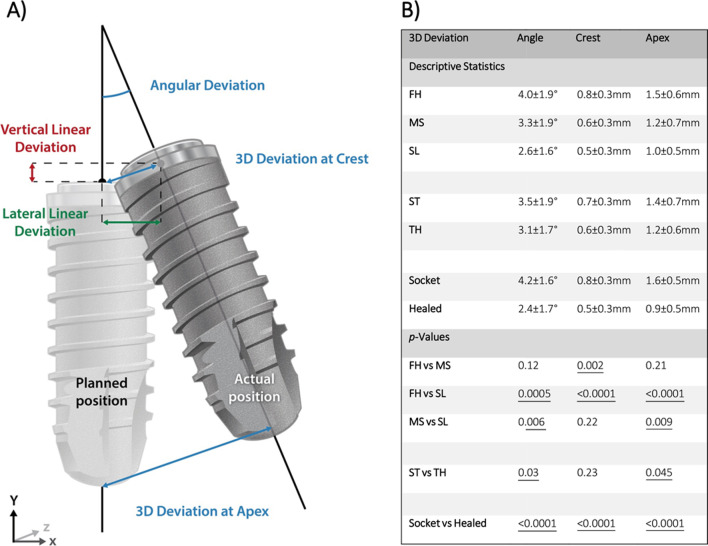
**A** Illustration depicting 3D angular, crestal, and apical implant deviation measurements. **B** Descriptive statistics (means ± standard deviation) and *p*-values of the 3D angular, crestal, and apical implant deviation for implant placement procedure (*FH* free-handed, *MS* manufacturer’s sleeve, *SL* sleeveless), implant system (*ST* Straumann, *TH* Thommen) and alveolar ridge morphology (*socket* fresh extraction socket, *healed* healed alveolar ridge). *p*-values < 0.05 displayed underlined

After the implants were placed, a post-operative CBCT scan of each study model was taken following the same baseline parameters (8 × 5 cm, 80 μm voxel size, 90 kVp, 1 mAs; 3D Accuitomo 170, J. Morita Corp, Osaka, Japan). The resulting DICOM files were imported to implant planning software (coDiagnostiX, version 10.5, Dental Wings Inc, Montreal, Canada). The pre- and post-operative DICOM files including the virtual pre-operatively planned implant position and final implant position were superimposed by manually matching at least five anatomical hard tissue landmarks (i.e., adjacent teeth) and using the software’s best-fit algorithm. Subsequently, the final implant position was obtained by the superimposition of an identical virtual implant in post-operative CBCT. The 3D angular and linear deviations between the final implant position and initial planning were measured automatically by the software’s algorithm at the most crestal and apical implant positions, as shown in Fig. 3. After a period of 3 months, the measurements were repeated for half of the sample to allow for the assessment of the intra-rater agreement.

### Statistical analysis

After the placement and deviation measurement of 36 implants in 6 Models, a sample size calculation was performed to detect the significance between factors in the procedure (FH, MS, SL) and alveolar ridge morphology (extraction socket, healed ridge) in at least 80% of cases. The primary outcomes of the sample size calculations were the angular, the crestal and the apical mean deviations (three outcomes). The power analyses were conducted as follows: the number of models was continuously increased by 1 (i.e., 6 implants). Using data from pilot study, 500 samples were then drawn and a three-way ANOVA was performed on this simulated data. Then, it was checked whether factors procedure and/or morphology (but not implant system) showed a significant main effect on the investigated outcome and resulted in a total of 18 models/108 implants. The measurements of the 36 implants used for sample size calculation were included in the final statistical analysis. All collected data were descriptively summarized by using mean/sd/min/Q1/median/Q3/max statistics and by showing box plots and tables. A non-parametric three-way ANOVA was conducted for each of the primary and secondary outcomes, with *p*-values < 0.05 statistically significant. The three-way ANOVA always assessed the above-mentioned factors, including up to two-way interactions. The intra-rater agreement was investigated for all measurement categories using the intra-class correlation coefficient [33]. All analyses were obtained using the statistics software R, version 4.0.2 [34].

## Results

### Study sample and intra-rater agreement

A total of 108 implants were included in this study. Fifty-four implants were placed utilizing the ST system (*n* = 18 FH, *n* = 18 drill-key system with MS, and *n* = 18 drill-key system with SL), and 54 implants were placed utilizing the TH system (*n* = 18 FH, *n* = 18 keyless system with MS, and *n* = 18 keyless system with SL) in sites with either healed alveolar ridge or extraction socket morphology. The correlation coefficients corresponding to the digital measurements for 3D angular, crestal, and apical deviations ranged from 0.86 to 0.99, reflecting good-to-excellent intra-rater agreement.

### Implant system

**Fig. 4:**
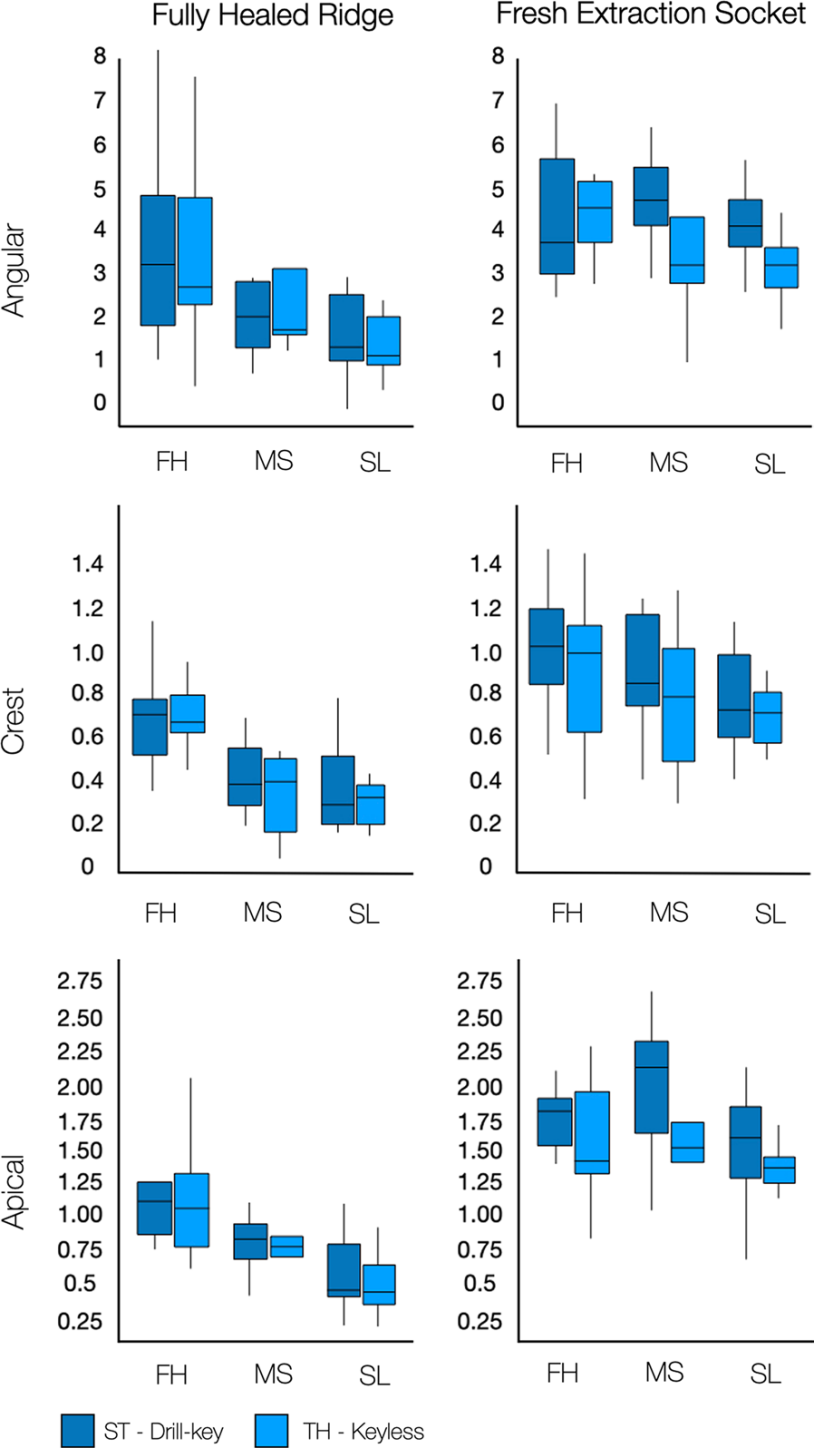
Box plots demonstrating 3D implant deviations (angular, at the crest and apex) when a free-hand technique, or sCAIS utilizing a manufacturer’s sleeve or sleeveless guide-hole designs were used in alveolar ridges with different morphologies. *FH* free-handed, *MS* manufacturer’s sleeve, *SL* sleeveless; ST: bone level 4.1 × 12 mm RC, Straumann AG; TH: element MC 4 × 12.5 mm Thommen SPI

Higher statistically significant 3D angular and apical mean deviations were observed when drill-key ST implants were placed compared to keyless TH implants (3.5 ± 1.9° vs 3.1 ± 1.7°, *p* = *0.03*; and 1.4 ± 0.7 mm vs 1.2 ± 0.6 mm, *p* = *0.045*) when extraction sockets and healed ridges were evaluated together. Nevertheless, no statistically significant difference was observed in mean deviations at the crest between drill-key ST and keyless TH implants (0.7 ± 0.3 mm vs 0.6 ± 0.3 mm; *p* = *0.23*). Descriptive statistics and representing box plots for implant system-related deviations are displayed in Figs. 3 and 4.

Drill-key ST and keyless TH implants were compared according to the alveolar ridge morphology. In healed ridges, drill-key ST implants showed angular, crestal, and apical mean deviations of 1.9 ± 0.9°, 0.4 ± 0.1 mm, and 0.7 ± 0.3 mm, respectively, as compared to keyless TH implants that showed mean deviations of 1.7 ± 0.8°, 0.3 ± 0.1 mm, 0.6 ± 0.3 mm. However, no statistically significant difference was observed between implant systems for angular (*p* = *0.629*), crestal (*p* = *0.373*), and apical deviations (*p* = *0.601*). Contrarily, in extraction sockets, the mean angular, crestal, and apical mean deviations for drill-key ST implants were 4.8 ± 1.6°, 0.8 ± 0.3 mm, and 1.8 ± 0.5 mm, while in the keyless TH implants mean deviations were 3.4 ± 1.6°, 0.7 ± 0.3 mm, 1.4 ± 0.4 mm. These results demonstrated higher implant position accuracy for the keyless TH implants in the angular (*p* = *0.02*), and apical deviations (*p* = *0.004*) but not the crestal region (*p* = *0.3*)*.*

### Implant placement protocol and surgical guide-hole designs

Implant placement following an FH protocol showed mean angular, crestal, and apical deviations of 4.0 ± 1.9°, 0.8 ± 0.3 mm, and 1.5 ± 0.6 mm. Implants placed utilizing MS showed mean angular, crestal, and apical deviations of 3.3 ± 1.9°, 0.6 ± 0.3 mm, and 1.2 ± 0.7 mm. Implants placed utilizing SL showed mean angular, crestal, and apical deviations of 2.6 ± 1.6°, 0.5 ± 0.3 mm, and 1.0 ± 0.5 mm. These results demonstrated that placement of implants in an FH manner had higher 3D deviations compared to sCAIS with MS and SL guide-hole designs. Descriptive statistics and representing box plots for deviations according to guide-hole designs and FH protocol are displayed in Figs. 3 and 4.

The post hoc pairwise comparisons showed higher statistically significant angular deviations between MS vs SL (*p* = *0.006*), and FH vs SL (*p* = *0.0005*). However, no statistically significant differences were observed between FH and MS (*p* = *0.12*). At the crest, statistically significant differences were observed between FH vs MS (*p* = *0.002*), and FH vs SL (*p* < *0.0001*), but not between MS vs SL (*p* = *0.22*). At the apex, statistically significant differences were observed between MS vs SL (*p* = *0.009*), and FH vs SL (*p* < *0.0001*), but not between FH vs MS (*p* = *0.21*).

### Surgical guide deterioration

No statistically significant differences in mean weight reduction were observed for different guide-hole designs between baseline and after implant placement. The mean weight reduction in SL sites was − 0.11 ± 0.17 mg, versus MS sites − 0.24 ± 0.23 mg (*p* = *0.06*). However, the calculation of volumetric material reduction revealed − 0.10 ± 0.15 mm^3^ material loss for SL vs − 0.03 ± 0.03 mm^3^ for MS sites due to the difference in densities of SL and MS material, indicating statistically significant deterioration between SL vs MS guide holes (*p* = *0.006*), with higher deterioration in the SL group (Fig. 2).

### Alveolar ridge morphology

Statistically significantly higher angular, crestal, and apical deviations (*p* < *0.0001*) were observed in implants placed in fresh extraction sockets than in fully healed alveolar ridges. Implants placed in fresh extraction sockets showed mean angular, crestal, and apical deviations of 4.2 ± 1.6°, 0.8 ± 0.3 mm, and 1.6 ± 0.5 mm. Contrarily, with implants placed in fully healed alveolar ridges, mean angular, crestal, and apical deviations were 2.4 ± 1.7°, 0.5 ± 0.3 mm, and 0.9 ± 0.5 mm. Descriptive statistics and representative box plots for alveolar ridge-related deviations are displayed in Figs. 3 and 4.

The interaction term analysis did not reveal significance between factors such as the procedure: guide-hole design, guide-hole design: implant system, and implant system: procedure. Descriptive statistics for each of the subgroups are shown in Table 1.

**Table 1:** Descriptive statistics of the crest, apex, and angular 3D implant deviation for each of the subgroups: alveolar ridge morphology, procedure, and implant system

Alveolar ridge morphology	Procedure	Implant system	Mean	SD	Min	Q1	Median	Q3	Max
3D deviation crest (mm)									
Healed ridge	FH	ST	0.72	0.26	0.37	0.53	0.70	0.77	1.13
Healed ridge	MS	ST	0.43	0.16	0.20	0.30	0.39	0.55	0.69
Healed ridge	SL	ST	0.36	0.21	0.17	0.21	0.30	0.52	0.78
Healed ridge	FH	TH	0.74	0.33	0.32	0.62	0.67	0.79	1.47
Healed ridge	MS	TH	0.35	0.19	0.06	0.17	0.40	0.51	0.53
Healed ridge	SL	TH	0.34	0.16	0.16	0.21	0.33	0.38	0.69
Extraction socket	FH	ST	0.99	0.27	0.52	0.84	1.01	1.18	1.45
Extraction socket	MS	ST	0.88	0.27	0.41	0.74	0.84	1.15	1.23
Extraction socket	SL	ST	0.77	0.25	0.41	0.60	0.72	0.97	1.12
Extraction socket	FH	TH	0.88	0.38	0.32	0.62	0.98	1.10	1.43
Extraction socket	MS	TH	0.76	0.31	0.30	0.49	0.78	1.00	1.26
Extraction socket	SL	TH	0.74	0.25	0.50	0.57	0.71	0.80	1.29
3D deviation apex (mm)									
Healed ridge	FH	ST	1.32	0.77	0.30	0.91	1.15	1.28	2.81
Healed ridge	MS	ST	0.80	0.24	0.43	0.74	0.87	0.98	1.12
Healed ridge	SL	ST	0.63	0.33	0.24	0.44	0.50	0.84	1.13
Healed ridge	FH	TH	1.29	0.71	0.66	0.81	1.10	1.35	2.81
Healed ridge	MS	TH	0.78	0.28	0.26	0.74	0.81	0.88	1.17
Healed ridge	SL	TH	0.57	0.25	0.24	0.40	0.49	0.68	0.95
Extraction socket	FH	ST	1.81	0.32	1.43	1.56	1.81	1.90	2.43
Extraction socket	MS	ST	2.01	0.55	1.09	1.68	2.13	2.32	2.69
Extraction socket	SL	ST	1.53	0.45	0.72	1.32	1.62	1.81	2.12
Extraction socket	FH	TH	1.62	0.46	0.87	1.36	1.43	1.95	2.29
Extraction socket	MS	TH	1.43	0.62	0.29	1.44	1.53	1.72	2.43
Extraction socket	SL	TH	1.43	0.20	1.18	1.28	1.40	1.47	1.77
Angular deviation (°)									
Healed ridge	FH	ST	3.83	2.33	1.10	1.90	3.30	4.80	8.20
Healed ridge	MS	ST	2.10	0.77	0.80	1.40	2.10	2.90	3.00
Healed ridge	SL	ST	1.63	1.05	0.00	1.20	1.40	2.60	3.00
Healed ridge	FH	TH	3.52	2.41	0.50	2.40	2.70	4.80	7.60
Healed ridge	MS	TH	2.19	0.80	1.30	1.70	1.80	3.20	3.20
Healed ridge	SL	TH	1.39	0.70	0.40	1.00	1.20	2.10	2.50
Extraction socket	FH	ST	4.52	1.70	2.50	3.10	3.80	5.70	7.00
Extraction socket	MS	ST	4.77	1.11	3.00	4.20	4.80	5.60	6.20
Extraction socket	SL	ST	4.00	1.38	1.20	3.70	4.20	4.80	5.70
Extraction socket	FH	TH	4.38	0.93	2.90	3.80	4.60	5.20	5.40
Extraction socket	MS	TH	3.54	2.04	0.50	2.90	3.30	4.40	7.50
Extraction socket	SL	TH	3.39	1.28	1.80	2.80	3.30	3.70	5.90

*FH* free-handed, *MS* manufacturer’s sleeve, *SL* sleeveless

ST: bone level 4.1 × 12 mm, RC, Straumann AG; Basel, Switzerland TH: element MC 4 × 12.5 mm Thommen SPI; Grenchen: Switzerland

## Discussion

This study compared the accuracy of keyless vs drill-key implant systems for sCAIS using two guide-hole designs compared to a free-handed protocol in two clinical scenarios simulating healed alveolar ridge or extraction socket morphologies. The findings of this study demonstrated higher accuracy in final implant positioning compared to pre-operative planned position for sCAIS with SL guide-hole designs, followed by sCAIS with MS guide-hole designs, and the FH protocol. Higher accuracy was also observed in healed alveolar ridges, and when a keyless implant system was used in fresh extraction sockets, as compared to the drill-key system. Finally, higher volumetric material reduction was observed in surgical guides with SL guide-hole design as compared to MS surgical guides. Therefore, H0_1,_ H0_2_ and H0_3_ were rejected.

To the best of the author’s knowledge, this is the first study evaluating drill-key ST and keyless TH sCAIS systems, with their respective drilling protocols. Statistically, significantly higher 3D angular and apical mean deviations were observed in the drill-key ST group, but no differences were observed at the crestal level between implant systems. When the alveolar ridge morphology was evaluated according to the implant system used, no statistically significant differences were observed between drill-key and keyless systems in healed ridges. Contrarily, statistically significantly fewer angular and apical deviations were observed in the keyless TH implant system for fresh extraction sockets. Therefore, these findings should be taken into consideration when immediate or early implant placement using sCAIS is planned after tooth extraction. The findings observed in this study agree with previous studies evaluating different implant systems, where differences in implant positional accuracy were found between and within manufacturers [17, 18]. The differences between systems could be explained by drilling system design and protocol since the implant macro-design is similar between both investigated implants. In the drill-key ST group, the use of a drill key may add some additional movement during the osteotomy and implant placement, as the drill key requires one additional gap, providing tolerances in-between the surgical components. These additional tolerances could affect the final implant position, compared to the TH keyless implant system (Fig. 2A, C).

Implants placed in an FH manner demonstrated higher 3D deviations as compared with sCAIS utilizing MS, and SL surgical guide designs. The results observed in this study are in line with previous systematic reviews, where the conventional approach involving free-handed osteotomy preparation, and implant placement via mental navigation demonstrated higher 3D deviations than sCAIS [8, 35]. Nevertheless, SL surgical guides showed fewer discrepancies in final implant position with respect to pre-operative implant position compared to MS guide-hole designs. This finding was also reported in previous studies [19, 20, 36]. The increased accuracy observed in this study with the use of SL guide-hole design compared to the MS guide-hole design could be explained by the differences in tolerance gaps, which are necessary for the installation, but potentially contribute to deviations of guide-related surgical components [37]. First, the gap between surgical guide and sleeve is eliminated for the SL compared to the MS guide-hole design, resulting in a reduced number of gaps. Second, the dimension of the gap between guide and drill-key (ST) or drill (TH) might be reduced for the SL guide-hole by printing it with smaller dimensions than the manufacturer’s sleeve. The reduced gap dimensions result in less tolerances and tighter fit of surgical components within the guide and might contribute to accuracy in sCAIS procedures. However, a minimum tolerance is needed to precisely install the surgical components within the guide hole. If tolerances are too small, the handling of the system might be negatively affected as corresponding components have too much friction or even do not fit into the guide hole. To control this technique-sensitive manufacturing process, a precise and predictable 3D printer and the selection of the appropriate printer-specific guide hole offset is necessary [20]. Moreover, the findings of this study indicate that the inclusion of the MS may not be necessary for sCAIS, and its elimination could improve the accuracy of the final implant position.

Assessment of the deterioration of the surgical guides demonstrated significantly higher mean volumetric material reduction for SL than MS guide-hole designs when using the keyless implant system. However, no statistically significant differences were observed in terms of mean weight reduction. Similarly, one previous study by Oh and colleagues did not find statistically significant differences in weight before and after implant placement in the surgical guides used, utilizing a different keyless implant system [36]. Nonetheless, the method of assessment did not consider different densities of materials used for MS-sites (stainless steel) and SL-sites (resin) as evaluated in this study. The volumetric reduction observed in this study makes it debatable whether the use of an SL guide-hole design for the keyless implant system should be used in clinical cases, as the metallic drill-integrated sleeve guidance may cause deterioration of the resin-based SL guide holes. However, even though minor mean volumetric reduction was observed, displacement of surgical guide material into tissues might cause biological adverse events, which have not been investigated so far. Therefore, the authors believe that the use of biodegradable surgical guide materials might be the first option to overcome this possible tissue interaction.

With respect to alveolar ridge morphology, implants placed in a fully healed ridge demonstrated higher accuracy in the final implant position as compared to fresh extraction sockets, independently of whether implants were placed using sCAIS or following a FH manner. Interestingly, angular deviations were similar for FH implant placement in extraction sockets and healed alveolar ridges, while sCAIS showed an almost twofold increase of angular deviation in extraction sockets compared to healed alveolar ridges. This difference might be due to the deflection of the drill by the oblique bony plate found in extraction sockets, with the center of rotation being located at the level of the guide hole for sCAIS systems as described previously [31]. Therefore, we believe that the accuracy of implant placement could be affected by the local bone density and architecture (i.e., the dense cortical bone should be expected to cause more pronounced deflections than trabecular bone). Closed guide holes limit the control of the deflected surgical drill and do not allow for corrections in the drill axis as compared to the use of open sleeves or FH implant placement. Our findings also agree with previous publications evaluating the accuracy of implant placement in this type of clinical scenario [30, 31]. However, our findings disagree with a retrospective study where no statistically significant difference was observed at the crest and angular deviations between immediate compared to a delayed implant placement protocol. Interestingly, the only statistically significant difference was observed at the apex, with the immediate implant placement group presenting higher accuracy than the delayed group [38]. Nevertheless, differences between studies could be explained by the differences in implant length, and patient-related factors, or by the retrospective nature of their study design, which does not allow the establishment of a causal relationship between confounding factors.

Finally, this in vitro study is not exempt from limitations. First, although the present design allowed the standardization of different variables (i.e., free drilling distance, surgical guide design, implant dimensions), the findings from this study should be interpreted cautiously as numerous factors (i.e., mouth opening, clinical scenario, surgical guide support) could affect final implant position in daily clinical practice. Second, sample size calculation was carried out for single-factor evaluation, being corroborated with less power for significance in the interaction-term analysis. Third, all implants were placed by one experienced operator. Different deviations might be found with other investigators according to their level of experience. Fourth, global digital evaluation of both implant systems makes it impossible to evaluate the origin of deviations (i.e., implant macro-design and/or design of the surgical components). Fifth, accuracy is a commonly used term in scientific literature that involves trueness (closeness to reference) and precision (closeness among data points) [39]. Although we have reported only data for trueness, it can be argued that the standard deviations presented in this study could be used as a surrogate parameter of precision. Further preclinical and/or clinical studies should evaluate the surgical drill design, implant system, and implant macro-design (i.e., implant body, length, diameter, thread design) on the accuracy of the final implant position. This could be particularly important in immediate implant placement scenarios since these factors could influence implant placement by achieving more primary/biomechanical stability at the apical part of the alveolar socket.

## Conclusions

Within the limitations of this study, it can be concluded that:

- The keyless implant system showed smaller 3D implant deviations than the drill-key system, with pronounced evidence in extraction sockets compared to healed alveolar ridge morphologies.
- sCAIS using a sleeveless surgical guide-hole design demonstrated higher accuracy in the final implant position than sCAIS with a manufacturer’s guide-hole design and freehand protocol.
- Higher volumetric material reduction was observed in sleeveless surgical guides as compared with guides including manufacturer’s sleeve.
- Fully healed ridges demonstrated higher implant positional accuracy than implants placed in fresh extraction sockets.

## Data Availability

The datasets generated during the current study are available from the corresponding author on reasonable request.

## Abbreviations

3D: Three-dimensional
ANOVA: Analysis of variance
CBCT: Cone-beam computed tomography
DICOM: Digital imaging and communications in medicine
HIG: Height of instrument guidance
FDD: Free drilling distance
FDI: Fédération Dentaire Internationale
FH: Free-handed
MS: Manufacturer’s sleeve
sCAIS: Static computer-assisted implant surgery
SL: Sleeveless
ST: Straumann AG
TH: Thommen Medical
